# Early vvECMO implantation may be associated with lower mortality in ARDS

**DOI:** 10.1186/s12931-023-02541-z

**Published:** 2023-09-26

**Authors:** Peter Rosenberger, Lisa Korell, Helene A. Haeberle, Valbona Mirakaj, Alice Bernard, Linyan Tang, Andreas Körner, Peter Martus, Michael Koeppen

**Affiliations:** 1 Department of Anesthesiology and Intensive Care Medicine, University Hospital Tübingen, Hoppe-Seyler-Straße 3, 72076 Tübingen, Germany; 2 Institute for Clinical Epidemiology and Applied Biometry, Faculty of Medicine, University of Tübingen, Tübingen, Germany; 3 University Hospital, Tübingen, Germany

**Keywords:** ARDS, ECMO, Inflammation, Therapy

## Abstract

**Background:**

Venovenous extracorporeal membrane oxygenation (vvECMO) is used to treat hypoxia in patients with severe acute respiratory distress syndrome (ARDS). Nevertheless, uncertainty exists regarding the optimal timing of initiation of vvECMO therapy. We aimed to investigate the association between number of days of invasive mechanical ventilation (IMV) prior to vvECMO implantation and mortality.

**Methods:**

In this retrospective observational study, we included patients treated at an academic intensive care unit with vvECMO for severe ARDS. The primary outcome was all-cause 28-day mortality. We conducted a multivariate logistic regression analysis to estimate the association between number of days of IMV prior to vvECMO implantation and mortality after adjustment for confounders.

**Results:**

Out of 274 patients who underwent ECMO for severe ARDS, 158 patients (median age: 58 years) with relevant data were included in the analysis. The mean duration of IMV prior to vvECMO was significantly shorter in survivors than in nonsurvivors [survivors median: 1; interquartile range: 1–3; non-survivors median 4; interquartile range: 1–5.75; p = 0.0001). Logistic regression showed an association between the duration of ventilation prior to vvECMO and patient mortality. The odds ratio for the all-cause 28-day mortality and in-hospital mortality was significantly reduced in patients who received vvECMO within the first 5 days of IMV.

**Conclusions:**

Early vvECMO implantation may be associated with lower mortality in ARDS.

## Background

Severe acute respiratory distress syndrome (ARDS) is a life-threatening condition, and severe inflammation of the pulmonary tissue is its hallmark [1]. The inflammatory response leads to lung parenchymal barrier dysfunction, resulting in protein-rich fluid influx into the alveoli [2, 3]. This thickening of the diffusion barrier reduces oxygen uptake into the blood, leading to hypoxic respiratory failure. Causal therapy includes broad-spectrum anti-infective treatment, prone positioning, neutral fluid balance and low-tidal-volume ventilation [4–[6]. These interventions have decreased the mortality rate of severe ARDS from 80% in 1967 to approximately 45% today [7]. However, the mortality rate of ARDS remains unacceptably high.

In cases where conventional therapeutic interventions fail to improve systemic oxygenation, extracorporeal membrane oxygenation (ECMO) can aid in treating hypoxia. This allows the care team to intensify conventional therapeutic interventions, such as further de-escalation of invasive ventilation to the point where no pulmonary ventilation is necessary, known as a lung rest strategy. Despite the potential advantages of venovenous ECMO (vvECMO) for patients with severe ARDS, several questions about this therapy remain unresolved [8, 9]; for example, there is still no consensus on the ventilation strategy that should be employed for patients with vvECMO [10]. Additionally, the timing of vvECMO initiation remains a subject of ongoing research. The RESP Score and the Preserve risk score have been used to show that a shorter duration of mechanical ventilation before ECMO initiation is beneficial for patient outcomes [11, 12]. However, in these studies, either the use of veno-arterial ECMO was included in the analysis and the generation of the predictive score [12] or only a crude point estimate regarding the duration of mechanical ventilation prior to ECMO therapy was given [12].

Therefore, in this study, we aimed to investigate the association of the exact number of days of invasive ventilation on mortality in patients who undergo vvECMO implantation. Using a multivariate analysis, we found that age and the number of days of mechanical ventilation prior to vvECMO were the most important risk factors associated with increased mortality. Logistic regression confirmed these findings. Our findings suggest that mortality could range from 27.4% (0 days of invasive ventilation prior to vvECMO) to 56.7% (7 days of invasive ventilation prior to vvECMO). These results emphasize the importance of early consideration of vvECMO in the management of ARDS, particularly in patients in whom conventional therapy fails.

## Material and methods

### Study population and ethics

Data were collected from medical records of the University Hospital of Tübingen. The Ethics Committee of the hospital (IRB# 692/2022BO2) approved the study and waived the need for informed consent, as patient anonymity was preserved.

### Study subjects

We screened patients treated in the Department of Anesthesiology and Intensive Care Medicine from January 2012 to December 2022. The analysis identified 274 patients who underwent ECMO for severe ARDS. We excluded 116 patients due to incomplete datasets. Most importantly, in these cases, the exact timepoint of intubation could not be determined from the medical records.

This resulted in a study population of 158 patients who met the inclusion criteria. The inclusion criteria were severe ARDS as defined by the Berlin definition, treatment at or transfer to the University Hospital of Tübingen, no ECMO for circulatory support during ICU stay, and implantation of vvECMO for respiratory failure.

**Fig. 1:**
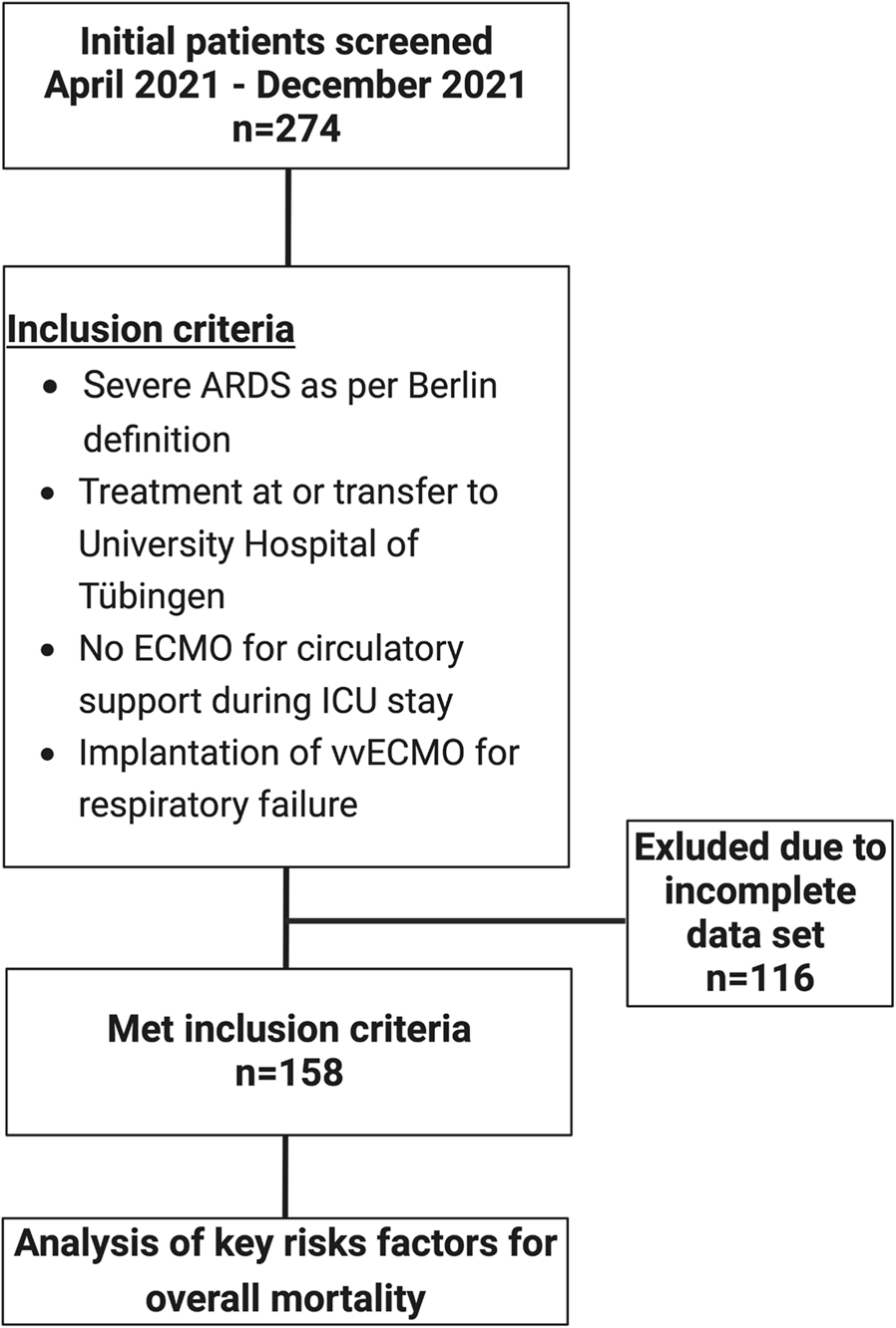
Patient selection strategy

ECMO initiation was guided by the Extracorporeal Life Support Organization (ELSO) guidelines [13]. The specific criteria for ECMO initiation included severe hypoxemia or severe hypercapnic acidosis after optimal conventional management, including a trial of prone positioning. However, given the rapidly changing and complex nature of the patients' conditions, there were cases where the exact timing of ECMO initiation deviated from the guideline recommendations. These deviations were based on individual clinical judgments considering each patient's specific condition [13] (Fig. 1).

### Data collection, statistical analysis, and model assessment

The primary outcome was all-cause 28-day mortality. We also analyzed data on the number of days on mechanical ventilation prior to vvECMO initiation, in-hospital mortality, laboratory values, and additional patient data, including age, sex, ICU admission and discharge dates, dates of intubation, and SOFA and APACHE II scores. The normal distribution of continuous variables was assessed using the Shapiro‒Wilk goodness-of-fit test. Variables are reported as the mean ± standard deviation or the median (interquartile range), as appropriate.

A series of statistical analyses, including univariate and multivariate logistic regression, were conducted to identify risk factors associated with increased mortality. Variables with a p value of less than 0.1 in the univariate analysis were selected for further multivariate analysis. The model's goodness-of-fit was assessed using the Hosmer‒Lemeshow test, and the area under the receiver operating characteristic (ROC) curve was calculated to evaluate its discriminative ability using the leaving one out correction.

Differences in variables between groups were evaluated using Student's t test, Mann‒Whitney U test, chi-square independence test, or Fisher's exact test, as appropriate. P values less than 0.05 were considered statistically significant. All statistical analyses were performed using JMP 16 (SAS Institute Inc., Cary, USA), Prism 9 (GraphPad Software Inc.), and SPSS Statistics for Windows v.28.0 (IBM Corp., 2020, Armonk, NY).

## Results

### Demographic data and patient characteristics

The flow diagram in Fig. 1 depicts the selection process employed in this study. Medical records were carefully screened to confirm the presence of an exact date of intubation for respiratory failure. Table 1 displays the fundamental characteristics of our study cohort. The majority of the patients were male, with a mean age of 51 (± 13) years. Despite a low 28-day mortality rate of 22.6%, 39.2% of patients did not survive until hospital discharge. All individuals included in the analysis received vvECMO due to severe ARDS, primarily caused by COVID-19 (56%). This was reflected in the median paO2/FiO2 of 73 mmHg prior to vvECMO implantation. The median interval between intubation and vvECMO cannulation was 1 day.

**Table 1:** Patient characteristics of the study cohort

	Total cohort (n = 158)	Survivors (n = 96)	Non-Survivors (n = 62)	P-value
Demographics				
Age—yr (mean ± SD)	51 ± 13	48 ± 13	55 ± 11	**0.0004**
Female sex—no. (%)	38 (24)	26 (27)	12 (19)	0.2671
Height—cm (mean ± SD)	1.75 ± 0.1	1.75 ± 0.1	1.75 ± 0.1	0.9214
Weight—kg (mean ± SD)	93 ± 22	95 ± 23	89 ± 20	0.0702
Body Mass Index (kg/m^2^) (IQR)	30 ± 6	30 ± 7	29 ± 6	**0.0487**
Overall mortality—no. (%)	62 (39.2)			
28-day mortality—no. (%)	36 (22.6)			
Days from intubation to VV-ECMO (IQR)	1 (1–4)	1 (1–3)	3.5 (1–6)	**< 0.0001**
Duration of vvECMO support (IQR)	19.5 (11–32)	19.5 (11–32)	19.5 (11–32)	0.5747
Renal replacement therapy no. (%)	84 (53)	42 (44)	42 (68)	**0.0035**^**#**^
On ICU admission				
SOFA (IQR)	8 (6–10)	8 (5–11)	9 (7–10)	0.6496
APACHEII (IQR)	18 (15 – 22)	18 (15–21)	19 (15.5–23.5)	0.4862
p_a_O_2_/FiO_2_–ratio (IQR)	76 (63–102)	74 (62–99)	83 (64–132)	0.0284
On day of VV-ECMO implantation				
SOFA (IQR)	9 (8–11)	9 (7–11)	10 (8–11)	0.0824
RESP-Score (IQR)	2 (0–4)	3 (1–4)	1 (-1–2)	**0.0001**
p_a_O_2_/FiO_2_–ratio (IQR)	73 (58–85)	73 (58–86)	72 (58–84)	0.9489
ARDS				
Primary ARDS n (%)	138 (87)			
Bacterial Pneumonia	23 (15)	18 (19)	5 (8)	0.0690^#^
Viral Pneumonia (other than COVID-19)	24 (15)	16 (17)	7 (11)	0.4889^#^
COVID-19 n (%)	88 (56)	49 (51)	39 (63)	0.1894^#^
Other causes	13 (8)	3 (3)	5 (8)	
Secondary ARDS n (%)	20 (13)	10 (10)	6 (10)	1.0000^#^
Comorbidities n (%)				
Lung disease (COPD, Asthma)	23 (15)	14 (14)	9 (14)	1.0000^#^
Coronary artery disease	8 (5)	4 (4)	4 (6)	
Active Smoker	22 (14)	14 (14)	8 (13)	0.8183^#^
History of malignancy (solid)	12 (8)	6 (6)	6 (10)	0.5411^#^
Autoimmune disease	6 (3)	3 (3)	3 (5)	0.6800^#^
Laboratory values on ECMO-Implantation				
Leukocytes (10^3^/µl; IQR)	12 (0.8–17.3)	12 (0.8–18.1)	12 (1.18–27.7)	0.4047
Thrombocytes (10^3^/µl; IQR)	211 (142–316)	228 (162–341)	194 (119–277)	**0.0322**
Creatinin (mg/dl; IQR)	1.1 (0.7–1.7)	1.0 (0.7–1.5)	1.2 (0.7–2.2)	0.2168
CRP (mg/dl; IQR)	18.5 (11–27)	18.3 (9–28)	18.5 (12.0–24.7)	0.9394
PCT (ng/ml; IQR)	1.51 (0.43–6.26)	1.35 (0.40–7.46)	1.7 (0.5- 4.0)	0.9985
Hemoglobin (g/dl; IQR)	10.9 (8.8–12.9)	11.9 (8.9–13.3)	10.1 (8.6–11.9)	**0.0069**

IQR: Interquartile range

^#^Fisher’s exact test

A notable characteristic was the prone positioning status before the initiation of vvECMO: 72.15% of the patients (114 individuals) were in a prone position, while 27.85% (44 individuals) were not. Upon closer inspection of the prone positioning status, our data revealed no significant difference in mortality outcomes between patients who were in a prone position before vvECMO initiation and those who were not (p > 0.05). This suggests that the prone positioning status before vvECMO initiation was not associated with in-hospital mortality in our cohort.

Our analysis further revealed that 41.8% of the patients did not meet the ELSO guidelines at the time of ECMO initiation. In this subgroup, the average duration of intubation before ECMO implantation was 4.62 days (95% CI 3.60–5.64), and the mean paO2/FiO2 prior to ECMO implantation was 93.97 mmHg (95% CI 88.37–99.57). This parameter was identified as the primary reason for not meeting the ELSO guideline criteria upon ECMO initiation. In contrast, among patients who did meet the ELSO guidelines at the time of ECMO initiation, the mean duration of intubation was shorter, at 1.75 days (95% CI 1.37–2.13), and the mean paO2/FiO2 prior to ECMO was lower, at 60.99 mmHg (95% CI 58.64–63.34).

Interestingly, no significant difference was found when comparing patients who met the ELSO guideline criteria for ECMO implantation between those who survived and those who did not (Chi-square = 1.836, p = 0.1755). Among the survivors, 37.5% met the ELSO guideline at ECMO implantation, whereas among non-survivors, this figure was slightly higher at 51.6%.

However, when considering adherence to the ELSO guidelines at the time of ICU admission, a statistically significant difference emerged (Chi-square = 4.402, p = 0.0359). The majority of survivors (69.3%) met the ELSO guideline at ICU admission, compared to only 37.1% of non-survivors.

### Mechanical properties of the respiratory system prior to ECMO implantation

**Fig. 2:**
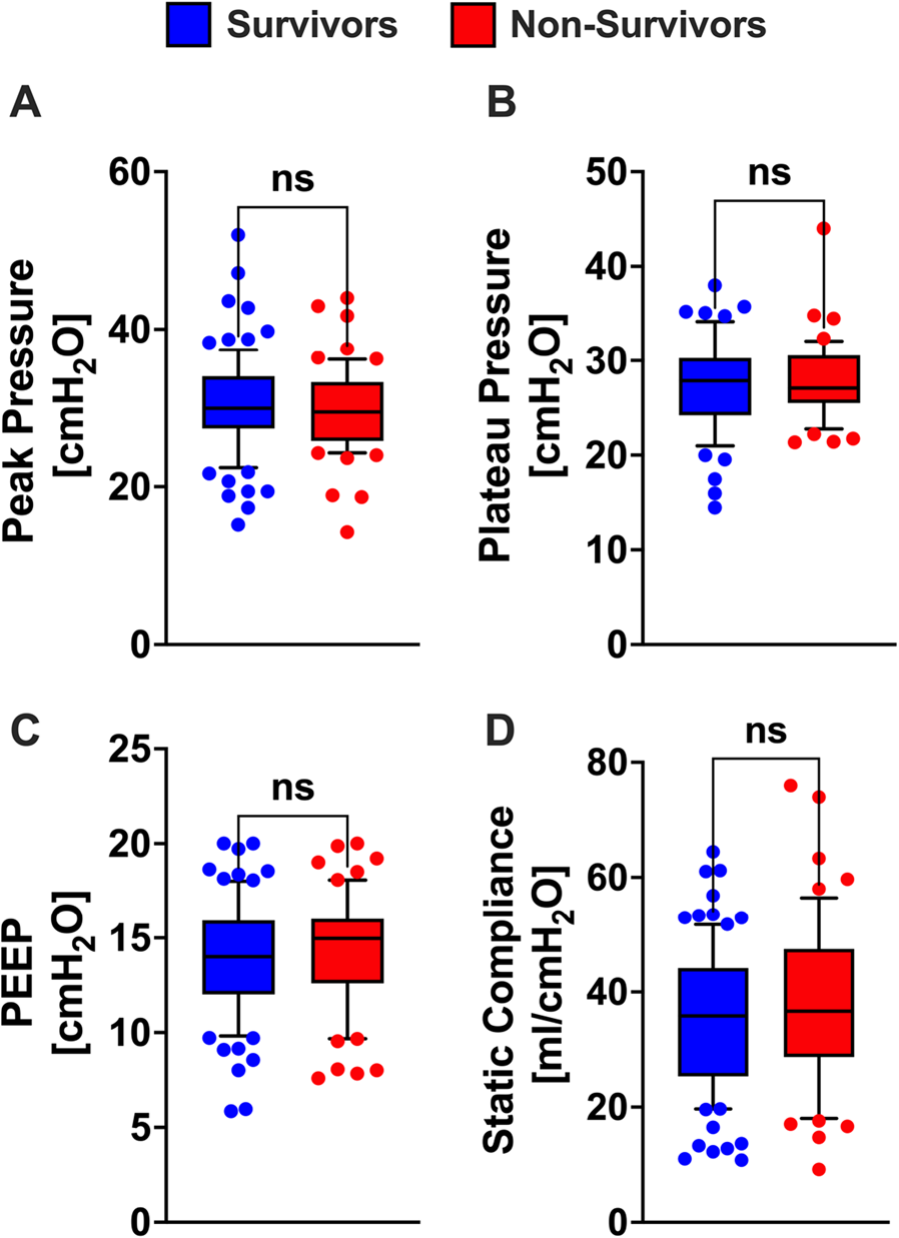
Comparative respiratory parameters between survivors and non-survivors prior to vvECMO implantation. **A** Peak inspiratory pressure: Survivors had a mean value of 35.17 ± 12 ml/cmH2O (n = 90), whereas non-survivors had 37.86 ± 14. (n = 59). **B** Plateau pressure: For survivors, the mean was 30.36 ± 6.10 cmH2O (n = 89), while non-survivors exhibited 29.68 ± 5.47 cmH2O (n = 60) **C** Positive end expiratory pressure (PEEP): Survivors showed a mean PEEP value of 13.88 ± 3.01 cmH2O (n = 89), whereas non-survivors had 14.27 ± 2.99 cmH2O (n = 60). **D** Static compliance: Survivors had a mean value of 20.80 ± 3.86 cmH2O (n = 90), while non-survivors exhibited 20.48 ± 4.01 cmH2O (n = 60). Data is presented median, whiskers extend from the 10th to the 90th percentile. All comparisons made using the Mann-Whitney-U-test

We investigated the association of pre-vvECMO mechanical respiration parameters on patient outcomes, hypothesizing that invasive ventilation beyond defined safe margins might influence mortality differences. Using the patients' data management system, we derived values for inspiratory peak pressure, plateau pressure, positive end-expiratory pressure, and the respiratory system's static compliance—parameters automatically recorded by the ventilators. Comparing these metrics between surviving and non-surviving patients revealed no significant differences in key ventilator variables, as illustrated in Fig. 2. Importantly, plateau pressures for survivors hovered around 30 cmH2O, while those for non-survivors were marginally lower at 29 cmH2O. Furthermore, per our institutional protocol, we did not use recruitment maneuvers in ARDS patients. These findings suggest that mortality discrepancies were not driven by variations in mechanical ventilation practices prior to vvECMO implantation.

### Factors associated with in-hospital mortality associated with vvECMO

We first conducted a univariate analysis to examine potential predictors of mortality, including age, days of invasive mechanical ventilation (IMV), platelet count, hemoglobin level, and immunosuppression. Age, days of mechanical ventilation, platelet count, hemoglobin level, and immunosuppression were all significantly associated with mortality in the cohort.

Subsequently, we performed a multivariate analysis incorporating factors associated with mortality in the univariate analysis. We found that only patient age and the number of days of mechanical ventilation before vvECMO were significantly associated with mortality. Additional to this analysis a leaving one out procedure was applied which leads to unbiased classification rates. The area under the curve for prediction of mortality was 0.72 (95% CI 0.64–0.80). The Hosmer Lemeshow statistic was 4.14 (df = 8, p = 0.84) and thus the fit of the model was very satisfactorily.

### Survivors and nonsurvivors and pre-ECMO invasive ventilation time

**Fig. 3:**
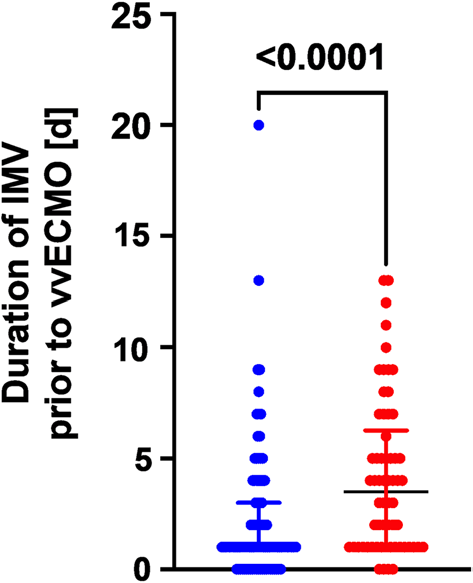
Difference in days of invasive mechanical ventilation (IMV) in survivors and non-survivors (in-hospital mortality). Survivors underwent IMV for a median of 1 day (interquartile range 1–3; n = 96), whereas non-survivors (defined based on in hospital mortality) underwent median IMV for 3.5 days (interquartile range 1–3; n = 62) (p < 0.0001). Compared by Mann–Whitney-U-test; data presented as median with interquartile range

We focused on the duration of IMV as a risk factor associated with mortality in patients undergoing vvECMO for severe ARDS. Our results suggested that a longer duration of IMV before vvECMO implantation was associated with increased mortality (Table 2). Figure 3 shows that survivors had significantly fewer mean days of IMV before vvECMO than nonsurvivors (p < 0.0001; survivors n = 96; nonsurvivors n = 62).

**Table 2:** Factors associated with overall mortality in vvECMO patients

	Univariate logistic regression		Multivariate logistic regression	
	Odds ratio (95% CI)	p-value	Odds ratio (95% CI)	p-value
Demographic/Complication				
Age (years)	**1.05 (1.02–1.08)**	**0.0004**	**1.06 (1.02–1.10)**	**0.0015**
Sex female	0.65 (0.30–1.40)	0.2690		
Body mass index	0.95 (0.9–1.01)	0.0708		
Renal replacement therapy	**2.7 (1.38–5.26)**	**0.0036**	2.17 (0.97–4.85)	0.0583
ICU admission				
SOFA	1.07 (0.85–1.35)	0.5474		
APACHEII	1.01 (0.95–1.07)	0.7724		
Admission paO2/FiO2	**1.01 (1.00–1.01)**	**0.0147**	1.01 (1.00–1.01)	0.0886
Etiology ARDS				
Bacterial pneumonia	**0.38 (0.13–1.1)**	**0.0705**	**0.23 (0.06–0.85)**	**0.0280**
Viral Pneumonia	0.74 (0.30–1.85)	0.5163		
COVID-19	1.63 (0.85–3.12)	0.1414		
Therapy prior to vvECMO				
Prone positioning	1.56 (0.75–3.25)	0.2312		
Days IMV prior to vvECMO	**1.20 (1.07–1.33)**	**0.0005**	**1.18 (1.03–1.35)**	**0.0092**
Day of vvECMO implantation				
White blood cell count (10^3^/µl)	1.0 (1.0–1.0)	0.2171		
Platelet Count (10^3^/µl)	**1.0 (0.99–1.0)**	**0.0147**	0.99 (0.99–1.00)	0.0819
Serum creatinine (mg/dl)	1.15 (0.84–1.58)	0.3766		
Hemoglobin (g/dl)	**0.82 (0.71–0.94)**	**0.0048**	0.92 (0.76–1.12)	0.4460
C-reactive protein (mg/dl)	1 (0.97–1.02)	0.7760		
Procalcitonin (ng/ml)	1 (0.98–1.01)	0.4615		
Comorbidities				
COPD	1.03 (0.27–3.82)	0.9595		
History of Asthma	0.96 (0.30–3.10)	0.9521		
Coronary artery disease	1.59 (0.38–6.59)	0.5271		
Immunosuppression	**5.96 (1.57–22.6)**	**0.0087**	2.76 (0.61–12.5)	0.1463

Boldface entries indicate a statistically significant association in either univariate or multivariate logistic regression analyses

We conducted a univariate analysis (binary logistic regression) to assess the association between the duration of IMV and 28-day or in-hospital mortality. Table 2 demonstrates that both parameters of mortality increased with increasing days of IMV before vvECMO implantation, suggesting that a shorter time between intubation and vvECMO implantation could be associated with lower mortality from severe ARDS.

**Fig. 4:**
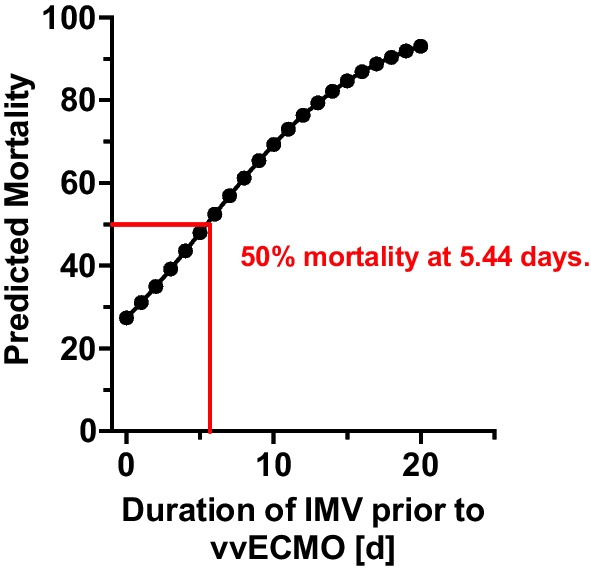
Inverse prediction of mortality based on days of IMV prior to vvECMO

We then performed a binary logistic regression investigating the correlation between days of IMV before vvECMO and in-hospital mortality. As shown in Fig. 4, the predicted values from the logistic regression of IMV duration before vvECMO were associated with increasing mortality. In a reverse prediction model based on the nominal logistic regression, mortality increased with every additional day of IMV before vvECMO (Table 3). Predicted mortality increased more than 50% with a duration of 5.4 days of IMV before vvECMO.

**Table 3:** Days of IMV prior to vvECMO and predicted mortality

Days of IMV prior to vvECMO	Predicted mortality (in %)
0	27.4
1	31.1
2	35.0
3	39.2
4	43.6
5	48.0
6	52.5
7	56.9
8	61.2
9	65.4
10	69.3
11	73.0
12	76.4
13	79.4
14	82.2
15	84.7
16	86.9
17	88.8
18	90.4
19	91.9
20	93.1

We used these results to define an "early vvECMO" group (IMV duration < 5 days) and a "late vvECMO" group (> 5 days of IMV duration). This approach provides a practical framework for clinicians to identify patients who could benefit from early vvECMO intervention. Our findings suggested that a shorter duration of IMV before vvECMO could potentially reduce mortality in patients with severe ARDS.

### Early versus late vvECMO

**Fig. 5:**
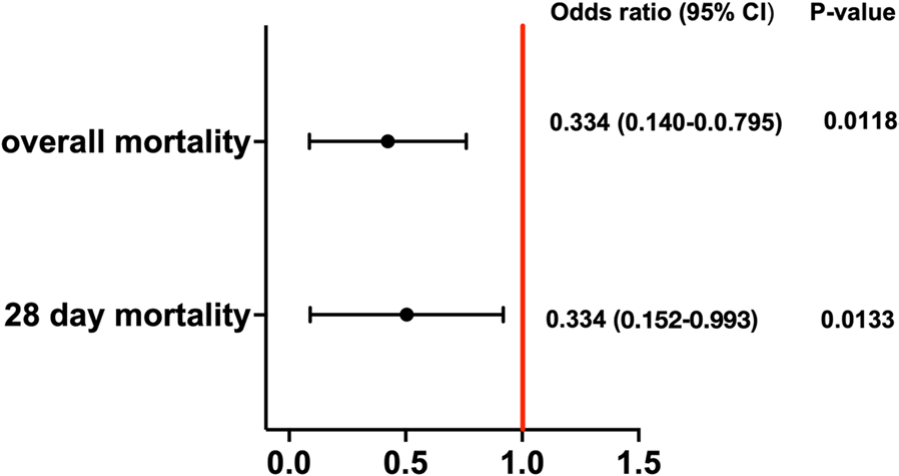
Odds ratio 28-day or in-hospital mortality with vvECMO < 5 days of IMV

Following the categorization of patients into "early" versus "late," we conducted an analysis of the odds ratio for patients who received vvECMO within 5 days of the initiation of mechanical ventilation (Fig. 5). Our findings revealed a significantly reduced odds ratio for in-hospital mortality in patients who received vvECMO within the first 5 days of invasive ventilation before vvECMO (Table 4). Therefore, early initiation of vvECMO was strongly correlated with lower in-hospital mortality rates.

**Table 4:** Effect of duration of IMV on mortality

	OR (95% CI)	p-values
28 day mortality	1.12 (1.01–1.25)	0.0233*
Overall mortality	1.20 (1.07–1.33)	0.0014*

*Compared by Wald Test

## Discussion

We aimed to examine the association between the duration of IMV prior to vvECMO therapy and in-hospital mortality in patients diagnosed with severe ARDS. Our findings suggested a positive correlation between the duration of IMV before vvECMO implantation and in-hospital mortality mortality. This association was also observed at 28-day and 90-day time points. Additional analysis was conducted by dividing patients into two groups: early vvECMO (defined as 5 days of mechanical ventilation or less) and late vvECMO. The results demonstrated that the early vvECMO group had a significantly lower mortality rate than the late vvECMO group. This supports the potential benefits of early initiation of vvECMO in reducing mortality in patients with severe ARDS.

Despite advances in care, ARDS remains a severe and potentially life-threatening syndrome that can affect individuals of all ages and socioeconomic backgrounds. Even with extensive research efforts over the last decades, mortality remains high, ranging between 30 and 50% depending on disease severity [14]. This number has remained relatively stable even amidst the COVID-19 pandemic. Therefore, vvECMO has been employed as a rescue therapy in patients unresponsive to conventional treatments. Several clinical studies and meta-analyses have suggested that vvECMO can decrease ARDS mortality, and various risk factors have been identified that are linked to patient mortality after vvECMO [8, 9, 11, 15, 16]. In this study, we performed a two-stage analysis of factors associated with in-hospital mortality in severe ARDS patients receiving vvECMO. In a multivariate analysis, we discovered that two primary patient variables correlated with mortality, namely, patient age and days of invasive ventilation before vvECMO. This aligns with prior studies, indicating that mortality in patients receiving vvECMO for severe ARDS increases with age [17]. We found that the number of days of IMV before vvECMO predicted mortality, which rose incrementally from day to day, reaching a predicted 50% mortality in 5.4 days. This is consistent with other studies that defined the RESP risk score to predict patient survival for vvECMO [11]. However, when calculating the RESP score, the investigators also included patients treated with veno-arterial ECMO. In contrast, our study cohort consisted exclusively of patients treated with vvECMO. Although we can partly confirm the results of the RESP score, this difference in the patients analyzed has to be considered.

Furthermore, the EOLIA trial, which is well known for its high rate of patients who switched from the control group to the vvECMO group, also reported increased mortality when IMV exceeded 7 days [9]. This finding differs from our study, in which we analyzed data from a single center and discovered a shorter period than 7 days. Therefore, while Schmidt and Combes provide a broader perspective on vvECMO mortality, our study examines our specific patient population more closely. The benefit of our investigation is that additional therapies such as ventilation strategy, prone positioning for vvECMO, and physical therapy are highly standardized at our institution, limiting the number of confounding factors that are inherent in retrospective studies. Recent data on mortality in vvECMO patients during the COVID-19 pandemic also support this notion, indicating that mortality rates may vary between institutions [18]. This study strongly suggests that vvECMO should only be carried out at specialized centers, which is corroborated by studies examining ECMO centers and their associated patient outcomes [19].

The results of Combes and Schmidt’s study, combined with our own findings, present a distinct perspective when compared to a recent retrospective analysis of 101 vvECMO patients [20]. This latter study didn't find a correlation between the duration of mechanical ventilation and vvECMO survival. While there are several overlapping aspects between our study and Hermann et al.'s, it's important to note that certain discrepancies might stem from differences in the datasets. For instance, Hermann et al. predominantly focused on COVID-19 patients. In contrast, half of our cohort examined ARDS from different causes. Furthermore, while Hermann et al. sourced their data from patients across six different ICUs in a relatively short time frame, we took a more longitudinal approach, gathering our data from one center over several years. This suggests that while Hermann et al. might have a more narrow, specialized focus aligning with other COVID-19 studies, our data offers broader insights, especially regarding the timing of vvECMO therapy. In essence, the two studies, rather than being contradictory, offer complementary perspectives due to differences in study population, time frames, design approaches (multi-center vs. single-center), and even the analytical methods used.

## Limitations

This report presents a retrospective analysis of a single-center study conducted over several years, which is a potential limitation. Patients were not randomized to a ventilation duration or strategy prior to vvECMO initiation. Importantly, data on the duration of IMV prior to vvECMO were missing for 158 patients, representing 42% of the potentially eligible cohort. This omission should be acknowledged as it may impact the generalizability of our findings. Nevertheless, our analysis involved more than 158 patients, providing some degree of generalizability for a rare event such as vvECMO implantation. The duration between intubation and vvECMO implantation varied widely, ranging from 1 to 20 days. This variability could introduce some bias, as patients may have already experienced respiratory failure for some time before vvECMO implantation, and longer intubation times are independently linked to increased ICU mortality. However, the observation that mortality increases incrementally implies that a better ventilation strategy may be applied once vvECMO is implanted. Another drawback of our study is that we did not examine the ventilation strategy. Therefore, in theory, patients who received vvECMO later may have been subjected to mechanical ventilation that could be considered unsafe.

## Conclusions

Our study underscores that postponing vvECMO implantation in patients with severe ARDS may correlate with an incremental rise in mortality. Factors such as the duration of intubation, vvECMO duration, and patient age emerged as potent mortality predictors. While our results provide valuable insights, they also underscore the pressing need for more comprehensive studies. Specifically, to conclusively determine the optimal timing for ECMO initiation, well-structured randomized controlled trials are imperative. Such trials will help in pinpointing a safe and effective interval between intubation and vvECMO implantation that does not exacerbate patient mortality.

## Data Availability

All data included in this study are available from the corresponding author on reasonable request.
